# Iron Deficiency Anemia in Inflammatory Bowel Diseases—A Narrative Review

**DOI:** 10.3390/nu13114008

**Published:** 2021-11-10

**Authors:** Dagmara Mahadea, Ewelina Adamczewska, Alicja Ewa Ratajczak, Anna Maria Rychter, Agnieszka Zawada, Piotr Eder, Agnieszka Dobrowolska, Iwona Krela-Kaźmierczak

**Affiliations:** Department of Gastroenterology, Dietetics and Internal Diseases, Poznan University of Medical Sciences, Przybyszewskiego Street 49, 60-355 Poznan, Poland; sledzinska.ew@gmail.com (E.A.); alicjaewaratajczak@gmail.com (A.E.R.); a.m.rychter@gmail.com (A.M.R.); aga.zawada@gmail.com (A.Z.); piotreder@ump.edu.pl (P.E.); adobzach@ump.edu.pl (A.D.); krela@op.pl (I.K.-K.)

**Keywords:** iron deficiency, IBD, dysbiosis, dietary factors, iron metabolism, microbiota, IDA

## Abstract

Inflammatory bowel disease (IBD), which includes Crohn’s disease and ulcerative colitis, is characterized by chronic inflammation of the gastrointestinal tract. IBD has been associated with numerous symptoms and complications, with the most common being iron deficiency anemia (IDA). Iron deficiency in IBD is caused by inadequate intake, malabsorption (including duodenal involvement and surgical removal), and chronic blood loss by mucosal ulcerations. Therefore, an appropriate diet should be enforced. Iron deficiency and iron supplementation have been associated with alterations to gut microbiota. IBD-associated anemia, in particular iron deficiency anemia, is associated with a significant decrease in quality of life and with clinical symptoms such as chronic fatigue, headaches and dizziness, reduced exercise tolerance, pale skin, nails, conjunctiva, and fainting. However, despite these numerous adverse symptoms, IDA remains undertreated. The European Crohn’s and Colitis Organisation (ECCO) guidelines state that patients should be monitored for anemia. Adequate treatment, whether oral or intravenous, should be implemented while taking into consideration C-reactive protein values (CRP), hemoglobin levels, and therapeutic response. It should be stressed that every case of anemia in IBD patients should be treated. Intravenous iron formulations, which are more superior compared to the oral form, should be used. There is a need to increase awareness and implementation of international guidelines on iron supplementation in patients with IBD.

## 1. Introduction

### 1.1. Inflammatory Bowel Disease

Inflammatory Bowel Diseases (IBD)—Ulcerative Colitis (UC) and Crohn’s Disease (CD) are a group of chronic inflammatory diseases of the gastrointestinal tract. They are characterized by the chronic and unpredictable course of the disease. Their multifactorial etiopathogenesis has not been clearly defined to date. They include, among others, immunological background, genetic, and environmental factors [1,2].

The highest incidence rates are observed in Europe and North America [3].

Today, more than 2 million Europeans and more than 2 million people in North America suffer from IBD, and the incidence of this disease is steadily increasing [4,5].

In a recent systematic review, the highest values were observed in Europe (UC 505 per 100,000 in Norway, CD 322 per 100,000 in Germany) and North America (UC 286 per 100,000 in the US, CD 319 per 100,000 in Canada) [4].

Clinically, both diseases are often manifested not only by symptoms of the gastrointestinal tract but also by complications of IBD involving other systems and organs, which may have a significant impact on the course and prognosis of the disease [6]. Patients with IBD are increasingly diagnosed at an early age. They must regularly take chronic treatment, face frequent hospitalizations due to exacerbations of the disease, and often surgeries, which can significantly affect their quality of life (QoL) and functioning. Often, these diseases contribute to the inability to live a full working life and other limitations. Therefore, the problem of IBD has become a significant global challenge for healthcare [7].

### 1.2. IBD-Associated Anemia

Anemia, which is considered the most common metabolic complication of inflammatory bowel disease, has been associated with an impairment in quality of life and cognitive function [8]. According to the World Health Organization (WHO), it can be defined as hemoglobin level <13 g/dL in men or <12 g/dL in non-pregnant women [8,9,10]. The European Crohn’s and Colitis Organisation (ECCO) guidelines classify anemia in IBD patients as iron deficiency anemia (IDA), anemia of chronic disease (ACD), and B12 or folic acid deficiency-associated anemia [8,11]. However, it is noteworthy that since IBD patients present with concomitant chronic intestinal bleeding, inflammation and ulcerations, impaired absorption, malnutrition, the toxic effects of medications, and undergo surgical procedures, the exact etiology of anemia cannot be determined. Pathogenesis is usually multifactorial [11]. Table 1 shows the most common pathomechanisms of anemia in IBD patients.

Despite numerous studies, the precise prevalence of anemia in IBD patients remains unknown. Most studies take into account selected groups such as hospitalized patients or those treated in high reference centers [12]. The occurrence of anemia ranges from 9–73% in outpatients and 32–74% upon admission [9,10,13]. The prevalence of iron deficiency in IBD-associated anemia is estimated at around 36–90% [14,15]. According to Woźniak et al., iron deficiency in newly diagnosed IBD patients is as high as 77.53% [6]. On the other hand, in the Inflammatory Bowel in South Eastern Norway cohort (IBSEN) study by Hoivik et al., which included 756 IBD patients at diagnosis and at the 1-, 5- and 10-year follow-ups, it was reported that 48.8% of newly diagnosed CD patients and 20.2% UC patients presented with anemia upon diagnosis. A total of 6.5% of patients had anemia more than 1 year prior to IBD diagnosis, while 13% of CD patients and 7.5% of UC patients presented with anemia 10 years from diagnosis [11,16,17]. In a systemic review and meta-analysis, including 2192 patients, Filmann et al. found that the overall incidence of anemia in IBD patients was 24%, and of severe anemia, 2.75%. Concomitantly, 57% of the anemic patients were found to be iron deficient [12]. In Italy, the prevalence of IBD-associated anemia amounts to 37%, with severe cases at 8.3%. Finally, Wilson et al. performed a systematic review and reported that in patients with CD, the occurrence of anemia ranges from 10.2–72.7%, and in UC, 8.8–66.6% [18].

Similar data from different studies report female predominance in the risk of IBD-associated anemia development [9,11,18,19].

Age has also been associated with a trend for a higher risk of anemia in IBD patients. Indeed, Woźniak et al. suggested that newly diagnosed, hospitalized patients aged from 18–25 years and >65 years presented with an elevated risk of developing anemia, as compared to those aged 25–65 years [6]. In accordance with this, the findings of Filmann demonstrated that male patients between 31–64 years had a lower risk of developing anemia. However, no such association was reported in female patients [12].

IBD disease activity has been linked with anemia. CD with structuring disease and extensive disease in UC are risk factors of anemia in IBD patients [12,20]. Elevated C-reactive protein (CRP) levels is an independent factor known to increase the prevalence of anemia in patients with IBD [6,19]. Moreover, Woźniak et al. demonstrated that ACD was predominant in CD in contrast to UC, where IDA was prevalent [6].

Numerous studies report that smoking decreases the risk for anemia in IBD patients. This is probably due to compensatory polycythemia caused by an increase in the consumption of carbon monoxide [8,21]. In contrast, the use of corticosteroid or immunomodulators result in an increased risk of anemia development [21].

## 2. Iron Metabolism

Iron is the basic building block of hemoglobin, ensuring the correct morphology and function of red blood cells. The human body contains approximately 3–5 g of iron. Most are found in red blood cell hemoglobin (2–4 g), in the spare form—ferritin or hemosiderin (1–1.5 g), myoglobin (100–300 mg), and enzymes (less than 100 mg) [22,23]. It occurs in the body in two forms. The first is the form that circulates in the blood and is transported by the transport protein ’transferrin’. This is essential to produce hemoglobin. The second form is the body’s iron store, where it is stored in a form bound to proteins—ferritin and hemosiderin. In the case of iron deficiency—insufficient supplementation, or malabsorption, there is a gradual deficiency of both forms of iron and, consequently, the production of red blood cells is impaired. As a result, fewer of them are created, they are smaller, and have a limited functionality. This contributes to a gradual deterioration of general health and the appearance of clinical symptoms of anemia.

In healthy adults, the daily loss of iron is about 1–2 mg/day in the form of shedding of the epithelial cells of the intestinal mucosa, biliary tract, urinary tract, and skin; and, additionally, in women in the form of menstrual blood loss. To ensure adequate iron management, the same amount of iron should be absorbed from food sources. Maximum iron absorption occurs in the duodenum and, to a lesser extent, in the proximal ileum [6,22,23].

Iron homeostasis is regulated by many mechanisms. Its content in the body is mainly controlled by its content in food, intestinal absorption, and recycling.

**Figure 1:**
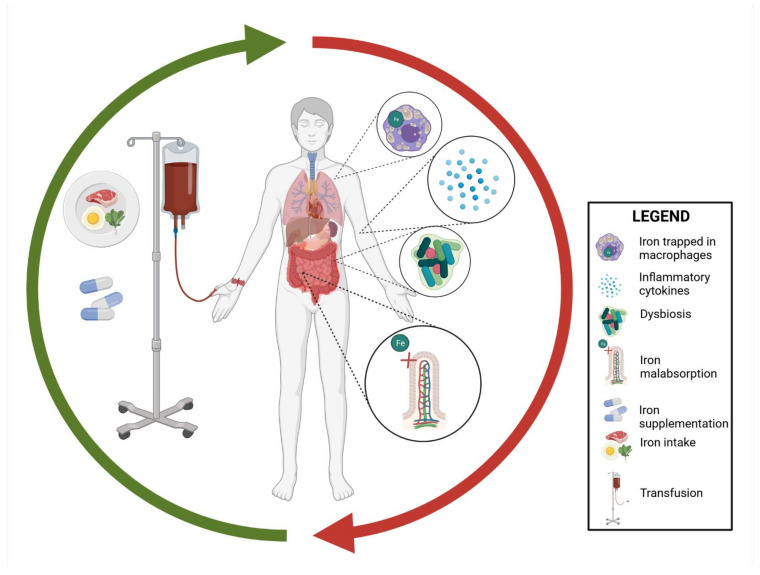
The effect of inflammation in IBD (inflammatory bowel disease) is mainly mediated by hepcidin [24]. Hepcidin is a peptide hormone produced in the liver. It plays a key role in regulating iron homeostasis. It is a direct inhibitor of ferroportin—a protein that transports iron beyond the cells that store it. The hepcidin–FPN (ferroportin) axis is considered to be the main regulator of iron homeostasis [24]. Inhibited ferroportin, present on enterocytes and macrophages, inhibits the transport of iron from enterocytes to the hepatic portal vein system, thus reducing iron absorption. Inhibited ferroportin leads to an inhibition of iron export, which is mainly found in the intestinal epithelium, macrophages, and hepatocytes. Consequently, the transport of iron absorbed by the intestines into the circulation and the release of iron from other cells is inhibited, which results in lowering the iron content in the serum. The inflammatory reaction significantly affects iron metabolism in the human body. The role of hepcidin explains the relationship between the immune response and iron metabolism [25,26].

In IBD, the most common cause of iron deficiency is a consequence of increased inflammation of the intestinal mucosa—its increased loss due to blood loss from the gastrointestinal tract, and malabsorption. In addition, the patients’ diet is also important, often leading to dietary restrictions [18]. Factors affecting iron levels in IBD patients are shown in Figure 1 below.

The conducted studies showed that in IBD patients, the increase in hepcidin was positively correlated with the increase in IL-6 and other pro-inflammatory cytokines, such as IL-1, IL-17, and tumor necrosis factor (TNF-alpha).

Hepcidin expression is downregulated by hypoxia, oxidative stress, IDA, hypoxia, and ineffective erythropoiesis, thus increasing iron availability [27].

TNF-alpha is the cytokine that plays the most important role in the pro-inflammatory processes in the pathogenesis of IBD. Its presence increases angiogenesis by activating macrophages and T cells, leading to direct damage to Paneth cells and intestinal epithelial cells [19].

Increased inflammatory cytokines have been shown to decrease mRNA expression of erythropoietin. The treatment with anti-TNF-alpha agents has been shown to improve iron deficiency by improving erythropoiesis, implicating a role for TNF-alpha in the development of IDA patients [19,24].

Iron is involved in the energy metabolism of all cells in the body, and most systems and organs. The most common clinical symptoms of moderate IDA include chronic fatigue, headaches and dizziness, reduced exercise tolerance, pale skin, nails, conjunctiva, and fainting [23,28].

In the case of greater deficiencies, the functioning of the circulatory system may be disturbed, with heart rhythm disturbances, systolic heart murmur, dyspnea at rest, and symptoms of angina. In addition, iron deficiency with concomitant anemia can lead to non-hematological symptoms [4] such as:

- Impaired cognitive performance;
- Thyroid hormone dysfunction;
- Catecholamine dysfunction;
- Increased risk of infection;
- Increased exposure to stress and depression in postpartum anemia;
- Disturbances in the functioning of neurotransmitters;
- Poorer outcomes of cognitive and motor development in children;
- Loss of libido, deterioration of sex life.

In patients with IBD, due to the adaptation of the organism, symptoms of iron deficiency are often non-specific and not very distinct. Additionally, the symptoms of the underlying disease may distort judgment here, due to their similar specificity. In older patients, it is associated with an increased number of hospitalizations and increased disability, a greater risk of falls, dementia, and a faster death. The impact of IDA on daily functioning and QoL has been shown to be significant. Chronic fatigue, in correlation with the manifestation of gastrointestinal symptoms, generates a great need for psychotherapeutic help in patients with IBD. In recent years, many studies have shown a high correlation of IDA with a significantly reduced overall QoL [22]. Therefore, it is believed that the correction of anemia in patients may be as important as the control and chronic treatment of IBD symptoms.

## 3. Iron in the Diet of IBD Patients

### 3.1. Iron and Its Requirements

Iron is an essential element for the human organism, as it participates in various metabolic processes, including hemoglobin and DNA synthesis. More than half of our iron is found in the hemoglobin in erythrocytes, 25% is stored (mostly in the liver, spleen, and bone marrow), and 15% is bound to myoglobin and a variety of enzymes. It should be noteworthy that the absorption of iron is low, ranging from 5% to 35%, and several dietary factors can enhance or limit its absorption [29]. On average, the adult organism stores around 1–3 g of iron, and a balance between dietary intake and iron loss is maintained. Around 1 mg of iron is lost every day from the desquamation of epithelia, and increases to about 2 mg per day during menstruation in women [30]. The proper intake of iron is especially vital, as both deficiency and excess can be harmful [31]. Iron deficiency mostly occurs in non-industrialized countries with poor dietary habits and high rates of chronic infections, but may also be present in industrialized countries [32]. It is also more prevalent among school-age children (almost 25.4% globally), adolescents, and premenopausal women due to the blood loss occurring during menstruation [33]. Moreover, iron requirements increase during pregnancy and breast feeding. Hepcidin—produced in the liver—is fundamental in iron homeostasis, and it has been observed that, in iron-deficient populations, its concentrations are usually low. Moreover, ‘anemia of inflammation’ due to increased hepcidin concentrations is also frequently observed among patients with acute or chronic inflammation or systemic illness [31]. Importantly, since the inflammatory blockade is major cause of iron deficiency, strategies reducing iron deficiency should also include limiting inflammation (also low-grade inflammation) and infections. Apart from iron-rich food products, iron can be provided from supplements or fortifications, although iron supplementation should be introduced in individuals with severe iron deficiency (or introduced universally in countries with high rates of iron deficiency). Among individuals with mild iron deficiency, food fortification should be considered as the preferred long-term strategy. Universal but uncontrolled iron supplementation can lead to an excess of iron and adverse events. Total iron requirements (95th percentile) according to the WHO are presented in the Table 2 [34].

### 3.2. Dietary Sources of IBD

Food sources of iron are divided into heme and non-heme. Non-heme iron is the oxidized form and must be reduced for transport across the intestinal epithelium. For this reason, non-heme iron has a lower bioavailability than heme [35]. Heme iron is present in animal products and non-heme both in animal and plant products [36]. Good plant sources of iron are beans, lentils, peas, spinach, nuts, and some dried fruits. Additionally, there are iron-fortified cereals and bread available in some countries [37]. The content of iron in various foods is presented in Table 3 [38].

It is vital to note that almost 80% of consumed iron is removed with feces, because there are many factors that may inhibit iron absorption [39]. For example, the first 40 mg of calcium in a meal does not inhibit iron absorption, but higher calcium concentration can limit iron absorption. Plant components in vegetables (including soya), coffee and tea, and calcium inhibit iron absorption. On the other hand, meat, fish, poultry, and ascorbic and organic acids increase iron absorption, especially non-heme [40]. However, both a high and low intake of vitamin C reduces iron absorption; around 25 mg of ascorbic acid (consider more if there are many iron inhibitors) should improve iron absorption [35]. Nevertheless, inhibiting and enhancing factors have a low impact on iron absorption, since a typical western diet attenuates their effects [41]. Dietary factors that may influence iron absorption are presented in Table 4.

Our proposed guidelines for IBD patients at risk of iron deficiency are as follows:

1. Patients should eat meat and fish. The best way to prepare meals is by boiling and roasting (due to the possible gastrointestinal symptoms);
2. Good sources of iron are also green vegetables; however, it should be noted that vegetables contain many compounds inhibiting iron absorption;
3. Legumes contain relatively high amounts of iron. Nevertheless, legumes are hard to digest and may be poorly tolerated by patients suffering from IBD;
4. Patients should reduce the intake of tea and coffee because some of the compounds present in coffee and tea inhibit iron absorption;
5. Patients should eat iron-rich plant products with food containing vitamin C, for example, spinach (also a source of iron) with lemon juice (vitamin C source), which will increase iron absorption.

## 4. Iron and Microbiota

Oral supplementation has been known to cause oxidative stress and mucosal damage, which aggravates inflammation and promotes carcinogenesis [43,44]. The chronic local inflammation characteristic of this disease inhibits its absorption. Unabsorbed in earlier sections, iron enters the colon, where it interacts with the intestinal microbiota. The release of lipocalin 2 (Lcn2) causes the sequestration of iron from bacterial siderophores. Studies conducted on healthy subjects are inconclusive. They show both positive and negative effects of iron supplementation on intestinal microflora and intestinal inflammation [45]. In comparison, no effect of oral iron supplementation on the severity of colitis has been demonstrated in patients with IBD [46]. In iron-deficient mice, it has been shown that oral iron administration can result in a decrease in beneficial microbiota and expansion of intestinal pathogens [47]. An increased abundance of the *Lactobacillus* family, *Enterobacteriaceae,* and *Enterococcus* was observed and a decreased quantity of *Bacteroides* and *Roseburia* members. A study by Constante et al. examined changes in the microbiome of mice given an iron-deficient diet compared to an iron-supplemented diet. Increased iron levels induced an increase in *Firmicutes* and had no effect on the number of *Bacteroidetes* and *Proteobacteria*. The number of each strain depended on the form of iron supplementation. Increased levels of *Parabacteroides* sp. (*Bacteroidetes* type) occurred with ferrous bisglycinate (FBG) as well as ferric ethylenediaminetetraacetic acid (FEDTA) supplementation [48]. In addition, differences in the SCFA content were also observed depending on the iron concentration. Low levels of butyrate and propionate were observed in rats during iron deficiency [49]. Iron is crucial for the replication of most bacteria. Some intestinal strains, such as *Bacteroides fragilis*, are highly dependent on heme (or its precursor, protoporphyrin IX) because they lack the ability to synthesize it [50,51]. Thus, both iron malabsorption and iron overload can significantly affect the ecosystem of heme-dependent intestinal bacteria. *Lactobacillus plantarum*, on the other hand, is a microorganism that does not require this element for its functioning [52]. This is confirmed by its presence in iron-limited environments (e.g., milk with high concentrations of lactoferrin).

Similarly, *Borrelia burgdorferi*, which has evolved in an iron-deficient environment, has replaced its presence in the metalloproteins that activate dismutase responsible for their virulence [53]. This facilitates infection in iron-free conditions.

In addition, the gut microbiota interacts with the body’s iron balance. Enteric bacteria of aerobic and anaerobic families, such as *Enterobacteriaceae, Streptomycetaceae*, and *Bacillaceae*, secrete siderophores or chelating compounds with a high iron affinity which collects iron from the environment [54]. Intestinal bacterial species can produce different iron-siderophore complexes and have other siderophore structures [54]. Iron absorption and retention decrease by about 25% in the absence of viable intestinal microflora, as confirmed by studies in rats and rabbits after antibiotic treatment [55,56].

## 5. Iron Supplementation in IBD

Despite symptoms such as fatigue, dizziness, headaches, dyspnea, tachycardia, syncope, shortness of breath, nausea, decreased QoL, and disorders of the immune system, IBD-associated anemia, especially IDA, remains undertreated [13,22]. Indeed, in a study performed in the USA, it was reported that 68.6% of patients were not further analyzed, and 25% were not treated [11]. Similar results were found in Switzerland, where treatment was implemented in 40% in private practices and 43% in public hospitals [11]. In a study conducted in 17 Greek IBD referral centers, only 18.1% were investigated for anemia [8]. According to a study involving 55 German GI centers, only 43.5% of anemic patients with IBD received treatment [10]. This low rate is astounding. One would expect that in our contemporary society, a basic ailment like anemia would not be overlooked. Since anemia affects the everyday life of patients and impairs their ability to work, treatment should be compulsory. The economic aspect should also be taken into consideration. The implementation of treatment will not only be associated with the resolution or decrease of symptoms but also with lower healthcare costs.

Regardless of the severity of anemia, treatment should be commenced in every IBD patient with IDA [9,22,57]. However, treatment of iron deficiency in patients with IBD who do not present with anemia is controversial. The benefits of such treatment in IBD have yet to be determined. The decision should be made individually [9,22].

The normalization of hemoglobin levels and iron stores requires a longer period of time [58]. However, an adequate therapeutic response is considered as a rise in hemoglobin of 2 g/dL in 4 weeks of treatment, a >30% increase in transferrin saturation, or serum ferritin >10 g/dL [9,13,57].

According to the ECCO guidelines, oral iron supplementation should be prescribed to IBD patients with normal CRP values and in clinical/endoscopic remission with mild IDA (hemoglobin >10 g/dL) [9,13,57]. Currently, two forms of oral iron preparations are available: a divalent Fe^2+^ and ferric form with sugar complexes Fe^3+^ [22,57,59]. Table 5 shows the most common iron formulations.

Since ferric preparations have been associated with a lower solubility, the use of ferrous formulations is more widespread [59]. However, a new ferric complex coupled with maltol has been investigated in a phase III trial. The authors suggest that the ferric iron has been correlated with a far better absorption rate, thus requiring smaller doses and decreasing the risk of adverse effects [9,22].

**Figure 2:**
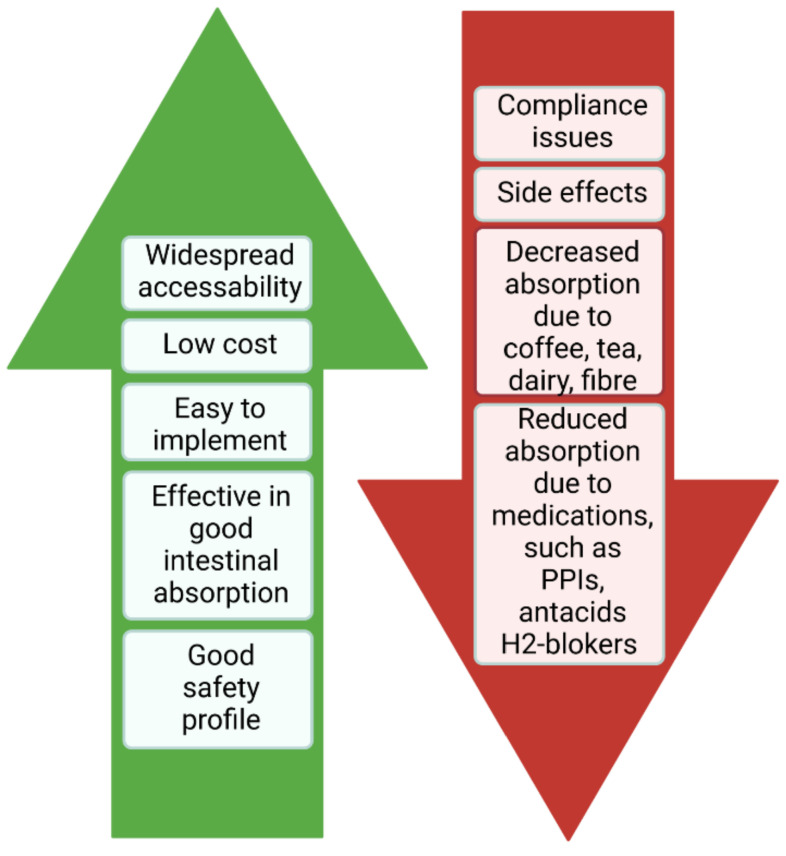
The advantages and drawbacks of oral supplements.

The advantages and drawbacks of oral supplements are shown in Figure 2 [13].

Although the precise dosage of oral iron supplementation has not been determined, a dose of 50–200 mg of iron daily is often prescribed [59]. However, since higher doses are co-dependent with lower compliance and more side effects, doses exceeding 100 mg daily are not recommended [9,13]. Indeed, low doses of 20–100 mg were found to be effective in the elderly and in pregnant women with anemia [9]. It should be noted that a meta-analysis in 2015 failed to find a correlation between the dosage of oral iron administration and the incidence of side effects [22]. It is recommended to prescribe oral iron once daily. It has been suggested that this might counteract the suppressing activity of hepcidin on iron absorption [58]. The use of oral iron preparations has been associated with numerous adverse reactions such as nausea, abdominal pain, constipation, and diarrhea [9,13,59]. Patients report the development of nausea and pain about 1–2 h after iron intake [59]. Although enteric-coated iron preparations have been linked with a lower occurrence of side effects, they are not absorbed as efficaciously [59]. Furthermore, only approximately 10–20 mg of ingested iron is absorbed from the gastrointestinal tract [2,59]. It is of common belief that the remaining unabsorbed iron causes mucosal harm and alters microbiota [9,58,59]. Indeed, according to animal trials with IBD, oral iron leads to exacerbation of IBD activity due to the production of pro-inflammatory cytokines [9,22,57]. A study in African children showed that oral iron supplementation was associated with an increase in fecal calprotectin, a rise in *Enterobacteria*, and a decrease in bifidobacterial in the intestinal microbiota [9,22,57]. Nonetheless, most evidence on the aggravation of IBD following oral iron supplementation was found in animal studies [9,57,59]. Convincing evidence in humans has yet to be made. Conversely, the effectiveness of oral iron supplementation has been reported in numerous studies [22]. In a study involving 78 IBD patients in remission with mild anemia, 89% presented with an adequate response. Only 5.1% were found not to tolerate this form of treatment [22]. Finally, due to the COVID-19 outbreak, the treatment approach has been altered. Virtual monitoring is on the rise, and planned appointments have been cancelled. As a result, oral iron supplementation has replaced other forms of anemia treatment [60].

ECCO guidelines indicate the use of intravenous (IV) iron in cases of severe anemia (hemoglobin level <10 g/L), inadequate therapeutic response, or intolerance of oral formulations [9,13,57,59]. The intravenous formulations comprise of a Fe^3+^ core and carbohydrate layer. The different preparations can be classified according to carbohydrate layers [9,13]:

1. 1st generation—high molecular weight iron dextran;
2. 2nd generation—low molecular weight iron dextran;
  a. Ferrous gluconate (Ferrlecit);
  b. Iron sucrose (venofer);
3. 3rd generation;
  c. Ferumoxytol;
  d. Iron carboxymaltose (Ferinject);
  e. Iron isomaltoside (Monover).

First-generation iron formulations have been associated with a higher rate of anaphylactic reactions or secondary effects [13,59]. The second generation has a greater safety profile. However, they are less stable and must be administered in small doses (about 200 mg), more frequently [59]. Indeed, higher doses or accelerated infusions cause the release of labile iron, which is inextricably linked to side effects [59]. Finally, third-generation preparations can be administered at a much faster rate (minimum 15 min), using high dosages [10,57,59]. Traditionally, the optimal dose of IV iron was calculated based on body weight and hemoglobin level according to the Ganzoni’s formula [2,9,13,27,59]: Total iron deficit [mg] = body weight × [(target Hb (hemoglobin) − actual Hb) × 2.4] + stored iron (500 mg).

However, several publications report that the iron need was undervalued [2,9,13] [27]. The FERGIcor trial published a simpler and more efficient scheme shown in Table 6. [2,9,57].

It should be emphasized that the evaluation of this scheme was limited to ferric carboxymaltose. However, its use has been adapted to other IV iron preparations [57].

Despite the adverse effects associated with IV iron such as itching, dyspnea, wheezing, myalgias, hypotension, tachycardia, stridor, nausea, dyspepsia, diarrhea, periorbital oedema, the possibility of hypophosphatemia, a potential risk of iron overload, and cardiac arrest, Chertow et al. published that the rates of life-threatening consequences were as low as 0.6, 0.9, 3.3, and 11.3 per million infusions for iron sucrose, ferric gluconate, high molecular weight iron dextran, and low molecular weight iron dextran, respectively [2,13]. Similarly, according to Wang et al., the risk of adverse reactions was lowest in iron sucrose as compared to dextran gluconate and ferumoxytol [2]. Patients should be monitored for at least half an hour following IV iron drips [16]. In a retrospective analysis by Akhuemonkhan and colleagues, it was found that only 1% of IBD patients developed adverse reactions, including anaphylactic shock [8]. The patients were administered IV iron formulations on the same day that they were treated with biologics [8,59]. It should be stressed that cross-reactions with antidextran antibodies and feruxomytol have been observed in vitro [2]. Therefore, patients who have presented with iron dextran intolerance should be particularly monitored when using these formulations. IV iron is considered more effective as compared to oral preparations [13]. It does not influence disease activity nor mucosal condition [13,57]. Four separate meta-analyses have shown the superiority of IV iron over oral iron in the treatment of IDA [2]. Indeed, higher ferritin levels have been reported upon IV treatment, resulting in a lower anemia recurrence [2]. In a meta-analysis including 669 IBD patients with IDA, it was noted that, due to side effects after oral iron supplementation, treatment had to be interrupted in 21% of patients [22]. In a systematic review and meta-analysis by Bonovas et al., a higher efficacy of IV iron products was observed [16,61]. This was in line with the findings of Nielsen et al. [16,58,59]. Although IV iron has been associated with a higher cost, it is estimated that using ferric carboxymaltose would allow a saving of between €263.40 and €87.10 compared to ferric gluconate, thus suggesting carboxymaltose as the economic choice [16]. Intravenous iron supplementation therapy is underrecognized by clinicians. Indeed, physicians tend to prefer the oral route, despite patients’ dissatisfaction and regardless of the superiority of intravenous formulations. Stein et al. showed that only 28% of patients were managed with intravenous iron [62]. According to Danese and associates, 72% of patients with implemented intravenous treatment were content, as opposed to 74% treated with the oral form [63]. We strongly believe that the use of intravenous iron should be encouraged. When taking into consideration the low treatment rates, it can be inferred that the formulation of guidelines is insufficient. We propose that workshops where doctors can practice the use of intravenous iron formulations should be developed. These should not be restricted to gastroenterologists and should be available to doctors with different specialties such as general practitioners, hematologists, and internal medicine specialists, among others. A better access to intravenous iron therapy should be made available. In Poland, intravenous iron treatment is scarcely available in out-patient centers. Indeed, in most cases, patients must be hospitalized. This should be altered. Our gastroenterological society should collaborate with the National Health Fund to improve access to IV iron treatment in ambulatory patient services. These centers should be fully equipped to enable such treatment. A proper method would be dedicating at least one day during the week for intravenous iron supplementation. More doctors and nurses should be employed during this day to ensure the safety of patients. Furthermore, gastroenterological societies throughout the world, whether local or international, should stress the importance of the treatment of anemia in IBD patients. Guidelines and information should be published for both doctors and patients. Gastroenterological societies should encourage the use of IV preparations in out-patient clinics. Patients should be motivated to undergo routine check-ups. IBD support groups should be formed where patients will have access to current treatment methods. This would improve the acceptance of IV supplementation by patients.

ECCO guidelines recommend monitoring IBD patients after IDA treatment every 3 months in the first year, and every 6–12 months thereafter [9,13]. Indeed, frequent recurrence of anemia is observed [9]. It is strongly believed that post IDA treatment, serum ferritin levels >400 ug/L are preventive for anemia recurrence [9,13]. Ferritin levels <100 ug/L, or hemoglobin <12 g/L in female patients and <13 g/L in men, are an indication for the reinitiation of IV iron [9,13]. However, Ali et al. stated that ferritin levels may not demonstrate iron availability in the first 8 weeks following treatment, and the response should not be assessed during that period [57].

## 6. Summary and Conclusions

To conclude, anemia is one of the most prevailing causes for hospitalization of IBD patients. Undoubtedly, it should constitute an important aspect of patient care because it is a frequent cause of a significant reduction in the QoL of patients, often young and professionally active people, making them incapable of work and normal functioning.

## Figures and Tables

**Table 1:** Pathogenesis of anemia in IBD patients [8,9,10,11].

Type of Anemia	Cause
IDA	• Iron loss from bleeding
	• Decreased iron intake from enterocytes
	• Impaired iron absorption
ACD	• Inhibition of erythropoiesis due to inflammatory cytokines
	• Iron trapped in macrophages
	• Dysfunction of iron transport
Vitamin B12 and foliate deficiency-associated anemia	• Malabsorption
	• Extensive small bowel resection
Drug-induced anemia	• Thiopurines, Sulfasalazine
	• Methotrexate

IDA—Iron deficiency anemia, ACD—anemia of chronic disease.

**Table 2:** Iron requirements and recommended iron intake for different diet bioavailability [34].

Group	Age (Years)	Total Requirements (95th Percentile, mg/Day)	Recommended Iron Intake for Different Diet Bioavailability (95th Percentile, mg/Day)	
			15% (High Bioavailability)	10% (Low Bioavailability)
Infants and children	0.5–1.0	0.93	6.20	9.30
	1–3	0.58	3.90	5.80
	7–10	0.63	4.20	6.30
	11–14	0.89	5.90	8.90
Females	11–14 ^PM^	1.40	9.30	14.00
	11–14	3.27	21.80	32.70
	15–17	3.10	20.70	31.00
	18+	2.94	19.60	29.40
Postmenopausal females	-	1.13	7.50	11.30
Lactating females	-	1.50	-	-
Males	11–14	1.46	9.70	14.60
	15–17	1.88	12.50	18.80
	18+	1.37	9.1	13.70

PM—premenarche.

**Table 3:** Content of iron in chosen food products [38].

Product	Iron Content (mg/100 g)
Pork liver	19
Cow’s milk	0.03
Herring	1.1
Lentils	8.6
Chocolate	0.3–0.5
Beef	3.1
Egg	1.3
Broccoli	1.1
Pasta	2.1

**Table 4:** Dietary factors that may influence the iron absorption [32,42].

Type of Iron	Factors Determining Iron Status	
Heme iron	Amount of dietary heme iron Contents of calcium in meal Food preparation	
Non-heme	Balance between enhancing and inhibiting dietary factors Amount of available non-heme iron	
	Enhancing factors	Inhibiting factors
Non-heme iron	Ascorbic acid	Phytate and phosphates
	Meat, fish, seafood	Iron-binding phenolic compounds
	Fermented vegetables or sauces (e.g., soy sauce)	Calcium
		Soya

**Table 5:** Available oral iron formulations [22,57,59].

Fe ^2+^	Fe^3+^
Ferrous fumarate	Iron protein sucinylate
Ferrous sulphate	Iron polymaltose complex
Ferrous gluconate	

**Table 6:** Estimation of iron dosage [2,9,57].

Hemoglobin g/dL	Body Weight < 70 kg	Body Weight > 70 kg
10–12 (women)	1000 mg	1500 mg
10–13 (men)	1000 mg	1500 mg
7–10	1500 mg	2000 mg
